# Characterization of bacterial communities on volcanic ash and pyroclastic deposits in Nishinoshima, Ogasawara Islands, Japan using 16S rRNA amplicon sequencing

**DOI:** 10.1128/mra.00647-25

**Published:** 2025-11-18

**Authors:** Yuka Homma, Tomoyuki Nakano, Kazuto Kawakami, Hideaki Mori, Takashi Kamijo, Tomoyasu Nishizawa

**Affiliations:** 1 Graduate School of Agriculture, Ibaraki University 12819, Ibaraki, Japan; 2 Seto Marine Biological Laboratory, Field Science Education and Research Center, Kyoto University 12918, Wakayama, Japan; 3 Hokkaido Research Center, Forestry and Forest Products Research Institute 57880, Hokkaido, Japan; 4 Japan Wildlife Research Center 91717, Tokyo, Japan; 5 Faculty of Life and Environmental Sciences, University of Tsukuba 13121, Ibaraki, Japan; 6 Green-Bio Technology Research Center, Ibaraki University 12819, Ibaraki, Japan; California State University San Marcos, San Marcos, California, USA

**Keywords:** bacterial community analysis, volcanic ash, pyroclastic deposit, Nishinoshima

## Abstract

We report bacterial community 16S rRNA amplicon sequencing results to provide insights into the formation process of terrestrial microbial communities on Nishinoshima, which was covered by volcanic ash and pyroclastic deposits from successive eruptions in 2020.

## ANNOUNCEMENT

The present Nishinoshima (140°52.5'E, 27°14.8'N), a volcanically active island surrounded by the sea, has been covered with fresh volcanic deposits by the 2020 huge eruption (1). The invasion and colonization of microbes play a crucial role in primary succession within volcanic deposits (2–4). The surface of volcanic ash and pyroclastic deposits (0–5 cm) was collected manually on-site using scoops in October 2016, May 2018, September 2019, and July 2022. In September 2021, the surface of deposits was collected using vacuum-equipped drones operated on the research vessel. Approximately 50 g of the collected samples were packed into sterilized plastic bags, respectively, and stored in a dark place.

Genomic DNA was extracted through a modified CTAB method (5, 6). The bacterial 16S rRNA gene region was amplified by using a 515F/806R primer pair [5′-GTGCCAGCMGGCCGCGGTAA-3′/5′-GGACTACHVGGGTWTCTAAT-3′; (7)]. All PCRs were carried out with 15 µL of Phusion High-Fidelity PCR Master Mix (New England Biolabs Inc., MA, USA), 2 µM of forward and reverse primers, and about 10 ng of template DNA. Thermal cycling consisted of an initial denaturation at 98°C for 1 min, followed by 30 cycles of denaturation at 98°C for 10 sec, annealing at 50°C for 30 sec, and elongation at 72°C for 30 sec. The final step was a 5 min extension at 72°C.

The sequencing was performed on the Illumina NovaSeq 6000 system (Illumina, CA, USA). Adapter-sequence removal was performed using Cutadapt v2.6 (8) (-overlap 3, -e 0.1) with default parameters. The processed sequences were subsequently analyzed using the mothur package v1.48.1 (9) following the standard operating procedure (10). Briefly, the 250 bp paired-end reads were merged (mothur command: make.contigs) and aligned (align.seqs) with the SILVA database v138 (https://www.arb-silva.de/) (11). Quality control was carried out (screen.seqs) and chimeric sequences were identified and removed using the VSEARCH algorithm (chimera.vsearch) (12). Taxonomic classification was performed using 16S rRNA reference (RDP) files v19 (https://sourceforge.net/projects/rdp-classifier/) (13) and sequences classified into non-bacterial taxa were removed (remove.lineage). Operational taxonomic units (OTUs) were clustered with 97.0% sequence identity and consensus classifications were obtained for each OTU (cutoff = 80). Then, taxonomic classification was performed again using RDP v19.

**Fig 1:**
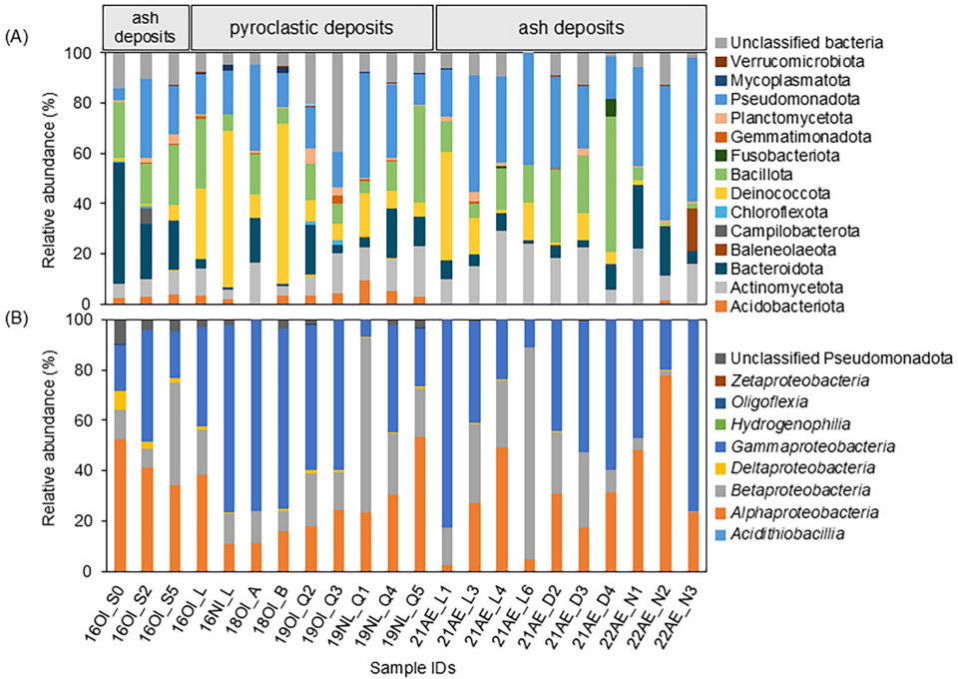
Relative abundance of bacterial phyla (**A**) and Pseudomonadota classes (**B**). The sample IDs are listed in the first column of Table 1. The reference to the phyla and class is shown on the right, respectively.

Table 1 shows the number of OTUs, ranging from 5,323 to 8,674, which were generated from 69,865 to 125,481 raw reads. According to the RDP analysis in the Nishinoshima volcanic deposits from 2016 to 2019, the dominant phyla were Pseudomonadota (20.8%), Deinococcota (18.9%), and Bacillota (16.6%) (Fig. 1A). In fresh volcanic ash deposits after the 2020 eruption, the phyla Pseudomonadota (27.8%), Bacillota (16.4%), and Actinomycetota (13.4%) became predominant (Fig. 1A). While the relative abundance within the phylum Pseudomonadota was dominated by *Alphaproteobacteria* (27.3%), *Betaproteobacteria* (26.7%), and *Gammaproteobacteria* (42.7%) in the volcanic deposits of former Nishinoshima, the relative abundances of these classes were 34.7%, 24.5%, and 40.3%, respectively, in fresh volcanic ash deposits (Fig. 1B).

**TABLE 1:** Summary of sample features and 16S rRNA gene-based sequence data of Nishinoshima

Sample features						
Sampling date/Sample ID	Deposit type	Sampling location/East longitude, North latitude	No. of raw reads	Coverage (%)	No. of OTUs	SRA accession no.
October 2016						
16OI_S0	ash	140°87.38', 27°24.57'	111,542	99.3	6,180	SRR32456516
16OI_S2	ash	140°87.38', 27°24.57'	107,343	98.1	8,352	SRR32456515
16OI_S5	ash	140°87.38', 27°24.57'	69,865	99.0	6,649	SRR32456504
16OI_L	pyroclastic	140°87.22', 27°24.67'	115,158	97.9	8,521	SRR32456501
16NI_L	pyroclastic	140°87.30', 27°24.61'	110,203	98.1	7,755	SRR32456500
May 2018						
18OI_A	pyroclastic	140°87.31', 27°24.65'	105,426	99.3	6,399	SRR32456499
18NS_B	pyroclastic	140°87.80', 27°24.40'	103,765	97.9	8,144	SRR32456498
September 2019						
19 Nl_Q1	pyroclastic	140°52.39', 27°14.72'	113,449	98.5	7,095	SRR32456497
19OI_Q2	pyroclastic	140°52.40', 27°14.75'	82,200	98.7	7,120	SRR32456496
19OI_Q3	pyroclastic	140°52.38', 27°14.77'	103,694	99.5	6,249	SRR32456495
19 Nl_Q4	pyroclastic	140°52.31', 27°14.88'	114,111	97.8	8,674	SRR32456514
19 Nl_Q5	pyroclastic	140°87.52', 27°14.49'	125,481	97.9	8,170	SRR32456513
September 2021						
21AE_L1	ash	140°88.17', 27°25.64'	113,691	99.4	5,958	SRR32456512
21AE_L3	ash	140°87.07', 27°24.62'	114,259	99.6	5,774	SRR32456511
21AE_L4	ash	140°88.80', 27°25.05'	103,465	99.6	5,733	SRR32456510
21AE_L6	ash	140°88.96', 27°24.87'	102,746	99.8	5,323	SRR32456509
21AE_D2	ash with seabird nest	140°87.13', 27°24.61'	103,218	99.6	5,877	SRR32456508
21AE_D3	ash	140°88.97', 27°24.88'	102,391	99.4	6,057	SRR32456507
21AE_D4	ash	140°87.19', 27°24.43'	103,511	99.5	5,941	SRR32456506
July 2022						
22AE_N1	ash	140°88.17', 27°25.64'	111,044	99.7	5,664	SRR32456505
22AE_N2	ash	140°88.21', 27°25.65'	105,517	99.7	5,568	SRR32456503
22AE_N3	ash	140°88.17', 27°25.64'	78,091	99.8	5,616	SRR32456502

## Data Availability

The 16S rRNA gene amplicon data sets are available through NCBI BioProject ID PRJNA1227021. Individual accessions for each sample are listed in Table 1.
